# Evaluating the relationship between alcohol consumption, tobacco use, and cardiovascular disease: A multivariable Mendelian randomization study

**DOI:** 10.1371/journal.pmed.1003410

**Published:** 2020-12-04

**Authors:** Daniel B. Rosoff, George Davey Smith, Nehal Mehta, Toni-Kim Clarke, Falk W. Lohoff

**Affiliations:** 1 Section on Clinical Genomics and Experimental Therapeutics, National Institute on Alcohol Abuse and Alcoholism, National Institutes of Health, Bethesda, Maryland, United States of America; 2 Medical Research Council Integrative Epidemiology Unit at the University of Bristol, Bristol, United Kingdom; 3 Section of Inflammation and Cardiometabolic Diseases, National Heart, Lung, and Blood Institute, National Institutes of Health, Bethesda, Maryland, United States of America; 4 Division of Psychiatry, University of Edinburgh, Royal Edinburgh Hospital, Edinburgh, United Kingdom; Imperial College London, UNITED KINGDOM

## Abstract

**Background:**

Alcohol consumption and smoking, 2 major risk factors for cardiovascular disease (CVD), often occur together. The objective of this study is to use a wide range of CVD risk factors and outcomes to evaluate potential total and direct causal roles of alcohol and tobacco use on CVD risk factors and events.

**Methods and findings:**

Using large publicly available genome-wide association studies (GWASs) (results from more than 1.2 million combined study participants) of predominantly European ancestry, we conducted 2-sample single-variable Mendelian randomization (SVMR) and multivariable Mendelian randomization (MVMR) to simultaneously assess the independent impact of alcohol consumption and smoking on a wide range of CVD risk factors and outcomes. Multiple sensitivity analyses, including complementary Mendelian randomization (MR) methods, and secondary alcohol consumption and smoking datasets were used. SVMR showed genetic predisposition for alcohol consumption to be associated with CVD risk factors, including high-density lipoprotein cholesterol (HDL-C) (beta 0.40, 95% confidence interval (CI), 0.04–0.47, *P* value = 1.72 × 10^−28^), triglycerides (TRG) (beta −0.23, 95% CI, −0.30, −0.15, *P* value = 4.69 × 10^−10^), automated systolic blood pressure (BP) measurement (beta 0.11, 95% CI, 0.03–0.18, *P* value = 4.72 × 10^−3^), and automated diastolic BP measurement (beta 0.09, 95% CI, 0.03–0.16, *P* value = 5.24 × 10^−3^). Conversely, genetically predicted smoking was associated with increased TRG (beta 0.097, 95% CI, 0.014–0.027, *P* value = 6.59 × 10^−12^). Alcohol consumption was also associated with increased myocardial infarction (MI) and coronary heart disease (CHD) risks (MI odds ratio (OR) = 1.24, 95% CI, 1.03–1.50, *P* value = 0.02; CHD OR = 1.21, 95% CI, 1.01–1.45, *P* value = 0.04); however, its impact was attenuated in MVMR adjusting for smoking. Conversely, alcohol maintained an association with coronary atherosclerosis (OR 1.02, 95% CI, 1.01–1.03, *P* value = 5.56 × 10^−4^). In comparison, after adjusting for alcohol consumption, smoking retained its association with several CVD outcomes including MI (OR = 1.84, 95% CI, 1.43, 2.37, *P* value = 2.0 × 10^−6^), CHD (OR = 1.64, 95% CI, 1.28–2.09, *P* value = 8.07 × 10^−5^), heart failure (HF) (OR = 1.61, 95% CI, 1.32–1.95, *P* value = 1.9 × 10^−6^), and large artery atherosclerosis (OR = 2.4, 95% CI, 1.41–4.07, *P* value = 0.003). Notably, using the FinnGen cohort data, we were able to replicate the association between smoking and several CVD outcomes including MI (OR = 1.77, 95% CI, 1.10–2.84, *P* value = 0.02), HF (OR = 1.67, 95% CI, 1.14–2.46, *P* value = 0.008), and peripheral artery disease (PAD) (OR = 2.35, 95% CI, 1.38–4.01, *P* value = 0.002). The main limitations of this study include possible bias from unmeasured confounders, inability of summary-level MR to investigate a potentially nonlinear relationship between alcohol consumption and CVD risk, and the generalizability of the UK Biobank (UKB) to other populations.

**Conclusions:**

Evaluating the widest range of CVD risk factors and outcomes of any alcohol consumption or smoking MR study to date, we failed to find a cardioprotective impact of genetically predicted alcohol consumption on CVD outcomes. However, alcohol was associated with and increased HDL-C, decreased TRG, and increased BP, which may indicate pathways through impact CVD risk, warranting further study. We found smoking to be a risk factor for many CVDs even after adjusting for alcohol. While future studies incorporating alcohol consumption patterns are necessary, our data suggest causal inference between alcohol, smoking, and CVD risk, further supporting that lifestyle modifications might be able to reduce overall CVD risk.

## Abstract 2

Daniel Rosoff and co-workers investigate impacts of alcohol and tobacco use on cardiovascular disease.

## Author summary

**Why was this study done?:**

- While some observational literature has reported a cardioprotective role of light-to-moderate alcohol consumption on cardiovascular disease (CVD) risk, more recent studies have failed to find evidence of a protective effect; however, these studies examined only a few CVD outcomes and risk factors.
- Randomized control trials (RCTs) are the gold standard for causal inference, but it is impractical and unethical to use RCTs to investigate the associations between alcohol consumption, tobacco smoking, and CVD endpoints. Therefore, uncertainty remains as to the causal nature of these relationships.
- Given the widespread consumption of alcohol and tobacco smoking, it is important to elucidate impact of each risk factor on a wide range of CVD risk factors and outcomes, which may help guide future clinical practice and research into treatment and prevention programs.

**What did the researchers do and find?:**

- We employed summary-level genetic data derived from more than 1.2 million combined study participants and conducted 2-sample single-variable Mendelian randomization (SVMR) and multivariable Mendelian randomization (MVMR) examining the impact of genetically predicted alcohol consumption and tobacco use on more than 50 CVD risk factors and endpoints.
- Genetically predicated alcohol and smoking were associated with increasing the risk for several CVDs, including stroke, myocardial infarction (MI), coronary heart disease (CHD), peripheral artery disease (PAD), atrial fibrillation (AF) and heart failure (HF), and hypertension.
- In MVMR, smoking retained a robust relationship with these CVDs, while alcohol consumption continued to increase risk for coronary atherosclerosis and hypertension.

**What do these findings mean?:**

- Using both SVMR and MVMR, which enables simultaneous assessment for genetically predicted alcohol and smoking, we find genetic evidence for an impact of alcohol consumption and smoking on CVD outcomes.
- We failed to find genetic evidence for a cardioprotective impact of alcohol consumption on CVD risk, which suggests that observational data identifying a cardioprotective role of alcohol consumption may have been due to confounding.

## Introduction

Cardiovascular disease (CVD) is a leading cause of mortality and morbidity with an estimated annual 17.9 million deaths worldwide [1]. Evidence shows that addressing modifiable lifestyle factors can prevent most CVDs [2]. Two highly prevalent and frequently co-occurring risk factors, alcohol consumption and smoking (affecting 2 billion and 1.1 billion people worldwide, respectively [2,3]), are associated with a considerable proportion of CVD mortality. For example, 1 in 10 CVD deaths are attributable to smoking [4], and observational studies strongly suggest that smoking increases the incidence of stroke, myocardial infarction (MI), and coronary heart disease (CHD) [5].

Observational studies show a complex relationship between alcohol consumption and CVDs, with some studies reporting light-to-moderate alcohol use associated with moderately reduced risk of MI [6] and CHD [7,8], and heavier alcohol consumption and binge drinking associated with increased stroke, MI, and CHD [8,9]. Similarly, meta-analyses and short-term trials suggest that alcohol consumption is associated with CVD risk factors including increased high-density lipoprotein cholesterol (HDL-C) [10]; however, the association of alcohol consumption with low-density lipoprotein cholesterol (LDL-C) and triglycerides (TRG) is less clear [11]. Despite its public health importance [10], nearly all human data evaluating the alcohol–CVD relationship are from conventional epidemiological studies [11]. Observational studies are subject to potential confounding and reverse causation, which makes causal inferences difficult [12]; many of the interventional studies or clinical trials examining the association between alcohol and CVD risk factors were small, failed to randomize different amounts of alcohol, and only observed short-term effects [10,13]. In sum, considerable debate remains regarding the association between alcohol consumption and CVD risk, whether alcohol has any cardioprotective effects, and potential biological pathways mediating the relationships [14,15].

One alternative strategy to investigate potential causal inference between an exposure and an outcome, in particular, when randomized control trials (RCTs) are not feasible, practical, or ethical, would be Mendelian randomization (MR) analysis [16]. MR analyses can be conceptualized as natural RCTs [17]. MR utilizes randomly inherited genetic variants, established before outcome onset, and therefore relatively independent of confounders, as instruments for an exposure to assess the causal effect of the risk factor exposure on a health outcome of interest [18]. Early MR studies using a single-SNP instrument found alcohol associated with increased HDL-C levels [13,15,19], lower TRG, and CHD risk [15], while a recent MR study using 512,715 Chinese adults found alcohol increased stroke risk but had no impact on MI [20]. Further, there is observational and genetic evidence linking alcohol consumption and smoking with approximately 85% of smokers drinking alcohol [21–23] and drinkers being 75% more likely than abstainers to smoke [24], suggesting estimates from previous single-variable Mendelian randomization (SVMR) analyses of alcohol, smoking, and CVDs may been impacted by failing to account for each behavior.

Multivariable Mendelian randomization (MVMR) is a recently developed method that allows for simultaneous assessment of separate but correlated exposures [25,26] by incorporating genetic variants from each risk factor into the same model [27]. MVMR has been recently employed to disentangle the direct effect for each risk factor not mediated by other correlated risk factors for a range of health outcomes, including a recent study using MVMR to compare the causal roles of lipids and apolipoproteins with coronary artery disease (CAD), which found the effects of LDL-C attenuated in MVMR models accounting for other lipids and lipoproteins, while the direct effect of apolipoprotein B (ApoB) remained, suggesting ApoB as a causal risk factor for CAD [28]. Assumptions underlying MR analysis require sufficient statistical power to perform the required sensitivity analyses [18]. The 2-sample SVMR and MVMR analyses used in this study—using summary-level data from separate genome-wide association studies (GWASs) for exposures and outcomes—allows for the use of complementary MR methods capable of investigating these assumptions [29].

Using a 2-sample MR design, we created genetic instruments for alcohol and smoking using summary statistics from publicly available, large-scale GWASs [22]. We used SVMR and MVMR methods to evaluate their impact on more than 50 CVD risk factors and disease events. The primary aim of this study was to use MR to comprehensively investigate both the total and direct relationships between alcohol consumption, tobacco smoking, and CVD across a wide range of CVD outcomes and risk factors.

## Methods

This study is reported according to the Strengthening the Reporting of Observational Studies in Epidemiology (STROBE) guideline (S1 STROBE Checklist).

### Protocol

This study did not have a prospective analysis plan. Initial discussion and project outline were discussed in May 2019 (i.e., use SVMR and MVMR methods to investigate the relationship between alcohol consumption, tobacco smoking, and CVD endpoints). Following peer review, we first repeated the SVMR and MVMR analyses using summary statistics for alcohol consumption and tobacco smoking derived from a newer and larger sample [30]. We expanded the number of CVD outcomes to include a more comprehensive evaluation of potential relationships and also leveraged the opportunity to include recently published CVD-related outcomes (heart failure (HF) [31] and cardiac magnetic resonance imaging (MRI) measurements of the left ventricle [32]) and the latest publicly available data release from the FinnGen cohort (Release 3, June 16, 2020) [33].

### Data sources

**Fig 1:**
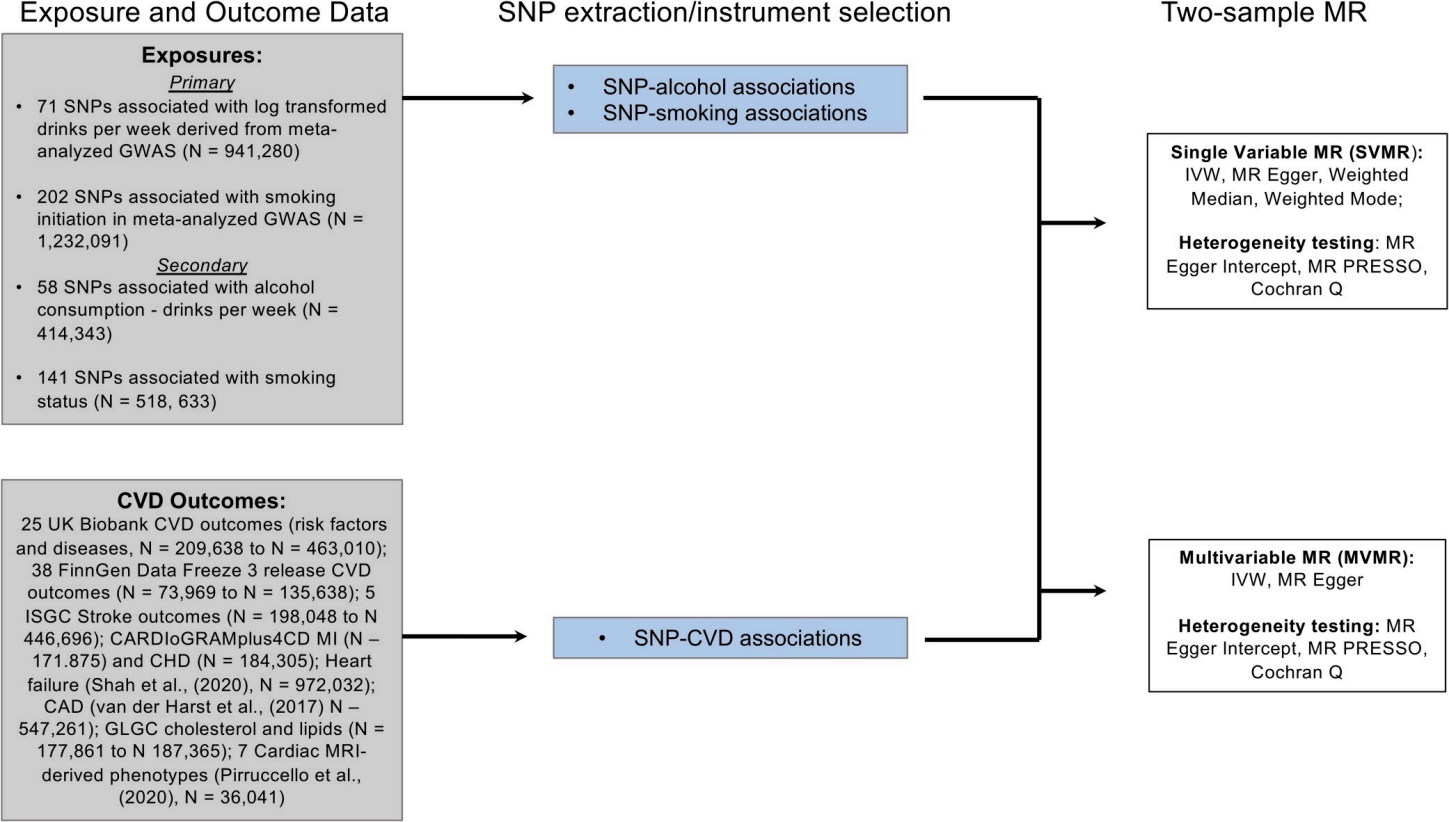
Study overview. CAD, coronary artery disease; CARDIoGRAMplusC4D, Coronary ARtery DIsease Genome-wide Replication and Meta-analysis (CARDIoGRAM) plus The Coronary Artery Disease (C4D) Genetics; CHD, coronary heart disease; CVD, cardiovascular disease; Dx., diagnosis; GLGC, Global Lipids Genetics Consortium; GWAS, genome-wide association study; IVW, inverse variance weighted; MR, Mendelian randomization; MVMR, multivariable Mendelian randomization; SNP, single nucleotide polymorphism; SVMR, single-variable Mendelian randomization.

We conducted single-variable (SV) as well as multivariable (MV) 2-sample MR analyses of alcohol consumption and tobacco use on CVD risk factors and outcomes using summary-level data from publicly available GWASs from similar populations of predominantly European ancestry of each of alcohol and tobacco use and CVD and disease risk factors (Fig 1, S1 Table). All studies comprising the GWASs have existing ethical permissions from their respective institutional review boards and include participant informed consent and included rigorous quality control. However, as this study was derived from summary-level data, ethics approval was not required for the present study.

Our primary genetic instruments for alcohol consumption and smoking were derived from the largest, predominantly white European ancestry, publicly available meta-analyzed (29 separate cohorts) GWAS summary association data (941,280 and 1,232,091 participants comprised the alcohol consumption and smoking data, respectively) [30]. Given the disparate methods for how weekly total alcohol consumption was measured across the cohorts (binned, normalized, etc.), the GWAS authors log-transformed the data, and therefore, the effect estimate is measured in log-transformed drinks per week) [30]. Our primary smoking instrument was constructed from the accompanying smoking initiation GWAS [30]. We included all SNPs associated with *P* < 5 × 10^−8^ and pruned all SNPs with the stringent pairwise linkage disequilibrum (LD) R^2^ > 0.001 (to assure statistical independence), giving instruments containing 71 SNPs for alcohol consumption and 202 SNPs for smoking initiation (S3 Table). We also used a second alcoholic drinks per week and second smoking status dataset for comparison to the primary alcohol consumption and smoking initiation analysis [22]. SNPs for alcohol use were extracted from a large GWAS meta-analysis of 414,343 individuals of European ancestry from the UK Biobank (UKB) prospective cohort study (collected across the United Kingdom from 2006 to 2010) with the phenotype “drinks per week” (DPW), constructed, for the UKB participants who indicated they drank “at least once or twice per week,” by aggregating the weekly intake of distilled spirts, beer and cider, red wine, white wine, champagne, and other alcoholic drinks (e.g., alcopops) (DPW: mean = 8.92 drinks, standard deviation (SD) = 9.30 drinks) [22]. For the UKB participants who indicated they drank “one to three times a month,” the phenotype was constructed by aggregating the monthly intake over all drink types and dividing by 4.

Smoking status SNPs were from the companion GWAS meta-analysis of 518,633 individuals of predominantly European ancestry (444,598 from the UKB prospective cohort study and 74,035 from the Tobacco, Alcohol and Genetics (TAG) consortium) [22]. We included all SNPs associated with genome-wide significance with the phenotype “ever smoker status” (a binary phenotype coded “1” if the individual reported he/she was a previous or current smoker and “0” if he/she had never smoked or only smoked once or twice). As with the primary instruments, we pruned the summary association data to exclude all SNPs with a pairwise LD R^2^ > 0.001, giving us 58 alcohol consumption SNPs and 141 independent SNPs associated with tobacco use for SVMR analyses (S3 Table).

For the MVMR analyses, we pruned the SNP instrument data to exclude any SNPs with a pairwise LD R^2^ > 0.001, leaving 117 and 179 SNPs, which were genome-wide significant in the GWASs of the primary and secondary alcohol consumption and smoking datasets, respectively. F statistics for the individual alcohol and smoking instruments were strong (>10, S3 Table). We were unable to calculate conditional F statistics to assess the strength of our MV instruments: SVMR statistical methods recently extended to 2-sample MVMR are appropriate only for nonoverlapping exposure summary-level data sources; when overlapping, the requisite pairwise covariances between SNP associations are determinable only using individual-level data [34].

### Cardiovascular risk factors

We used summary statistics from the GWAS meta-analyses for lipid levels—HDL-C, LDL-C, total cholesterol (TC), and TRG (measured in SD units (mg/dL))—in 37 cohorts comprising 188,577 persons of predominantly European ancestry (excluding persons known to be on lipid-lowering medications or women known to be pregnant) [35]. For comparison to the Global Lipids Genetics Consortium (GLGC) lipid panel and extension to other lipoproteins, we also included the 2019 release of the Neale Lab UKB GWAS (automated diastolic and systolic blood pressure (BP) measurement) and their biochemistry panel (HDL-C, LDL-C direct, TRG, apolipoprotein A (ApoA), ApoB, and lipoprotein A) [36]. We also included the recently released summary data for cardiac MRI in 36,041 UKB participants who had MRI data (of the 39,298 total) but did not have incident or prevalent HF, hypertrophic cardiomyopathy, or CAD prior to MRI collection [32].

### Cardiovascular disease events

Detailed sample information is available in S1 Table. We used summary statistics from the first Coronary ARtery DIsease Genome-wide Replication and Meta-analysis (CARDIoGRAM) plus The Coronary Artery Disease (C4D) Genetics (CARDIoGRAMplusC4D) GWAS meta-analyses for CAD (60,801 cases; 123,504 controls) and MI (CAD subgroup: 43,676 cases; 128,199 controls) in 48 cohorts in up to 184,305 persons of mixed ancestry (approximately 15% non-European ancestry, including Chinese, Indian Asian, South Asian, Lebanese, African American, and Hispanic American ancestry) [37]. We also included a more recent, larger CAD dataset comprised of the CARDioGRAMplusC4D and the UKB (*N* = 547,261: 122,733 cases and 424,528 controls) [38]. Atrial fibrillation (AF) summary data were obtained from meta-analyzed GWAS including 1,030,836 (60,620 cases and 970,216 controls) participants from the Trøndelag Health Study (HUNT), deCODE, Michigan Genomics Initiative (MGI), DiscovEHR, UKB, and Atrial Fibrillation Consortium (AFGen) studies [39]. HF summary association data from another meta-analyzed GWAS from 28 consortiums included 47,309 cases and 930,014 controls [31].

Summary-level association data from the International Stroke Genetics Consortium (ISGC) MEGASTROKE study were used as the primary stroke outcome data [40]. Samples were derived from predominantly European cohorts [40] and 5 stroke outcomes (stroke, ischemic stroke, larger artery atherosclerosis, small-vessel stroke, and cardioembolic stroke) with sample sizes ranging from *N* = 198,048 to *N* = 446,696. Summary statistics for 16 CVD outcomes from the Medical Research Council Integrative Epidemiology Unit (MRC-IEU) UKB GWAS Pipeline [41] (generated using the PHEnome Scan Analysis Tool (PHESANT) [42]) and the Neale Lab UKB GWAS [36] are listed in S1 Table. Data were derived from electronic health records (EHRs), hospital codes, and self-report of white European ancestry participants in the UKB. Detailed description regarding PHESANT and Neale Lab GWAS methods is available in the original publication ([42] and http://www.nealelab.is/uk-biobank, respectively).

We also leveraged the FinnGen Datafreeze 3 release (publicly available June 16, 2020) [33] to expand the number of CVD outcomes (in total, 41 CVD outcomes plus 16 CVD risk factors (circulating lipids, BP, and MRI-derived left ventricle function)) (S1 Table). Detailed documentation is provided on the FinnGen study website (https://finngen.gitbook.io/documentation/). FinnGen is a public–private partnership incorporating genetic data for 1,801 disease endpoints from Finnish biobanks and Finnish health registry EHRs [33]. FinnGen Datafreeze 3 included only European ancestry, and samples sizes ranged from *N* = 73,969 (cardiac arrest) to *N* = 135,638 (ischemic heart disease, hypertensive diseases, and hypertrophic cardiomyopathy). In addition to extending the number of CVDs evaluated, FinnGen data were also used to employ a discovery-replication type approach to the several of the CVD outcomes available in both the UKB or independent consortium CVD outcomes. These outcomes were AF, HF, MI, CAD, peripheral artery disease (PAD), atherosclerosis, stroke, stroke including subarachnoid hemorrhage (SAH), and stroke excluding SAH.

### Statistical and sensitivity analyses

For SVMR, we used inverse variance weighted MR (IVW MR) (SV weighted linear regression) along with the complementary MR–Egger, weighted median, and weighted mode methods to assess the evidence of the causal effects of each of alcohol and smoking initiation on CVD risk factors and disease outcomes and to detect the sensitivity of the results to different patterns of violations of IV assumptions [18] as consistency of results across methods strengthens an inference of causality [18]. For MVMR, we used an extension of the IVW MR method, performing MV-weighted linear regression (variants uncorrelated and random effects model) with the intercept term set to 0 [26,43]. We used an extension of the MR–Egger method to correct for both measured and unmeasured pleiotropy [44].

To evaluate heterogeneity in instrument effects, which may indicate potential violations of the IV assumptions underlying 2-sample MR [45], we used the MR–Egger intercept test [45], the Cochran heterogeneity test [46], and MV extensions thereof [43,44]. The Mendelian randomization pleiotropy residual sum and outlier (MR-PRESSO) global test and MV extension thereof [47] were used to facilitate identification and removal of outlier variants in order to correct potential directional horizontal pleiotropy and resolve detected heterogeneity. For the SVMR, we used the Steiger directionality test to test the causal direction between the hypothesized exposure and outcomes [48]. Analyses were carried out using MendelianRandomization version 0.4.1, TwoSampleMR version 5.3 [18], and MR-PRESSO version 1.0 [47] in the R environment version 3.6.3 (The R Foundation for Statistical Computing, Vienna, Austria).

### Reported results and interpretation of findings

**Fig 2:**
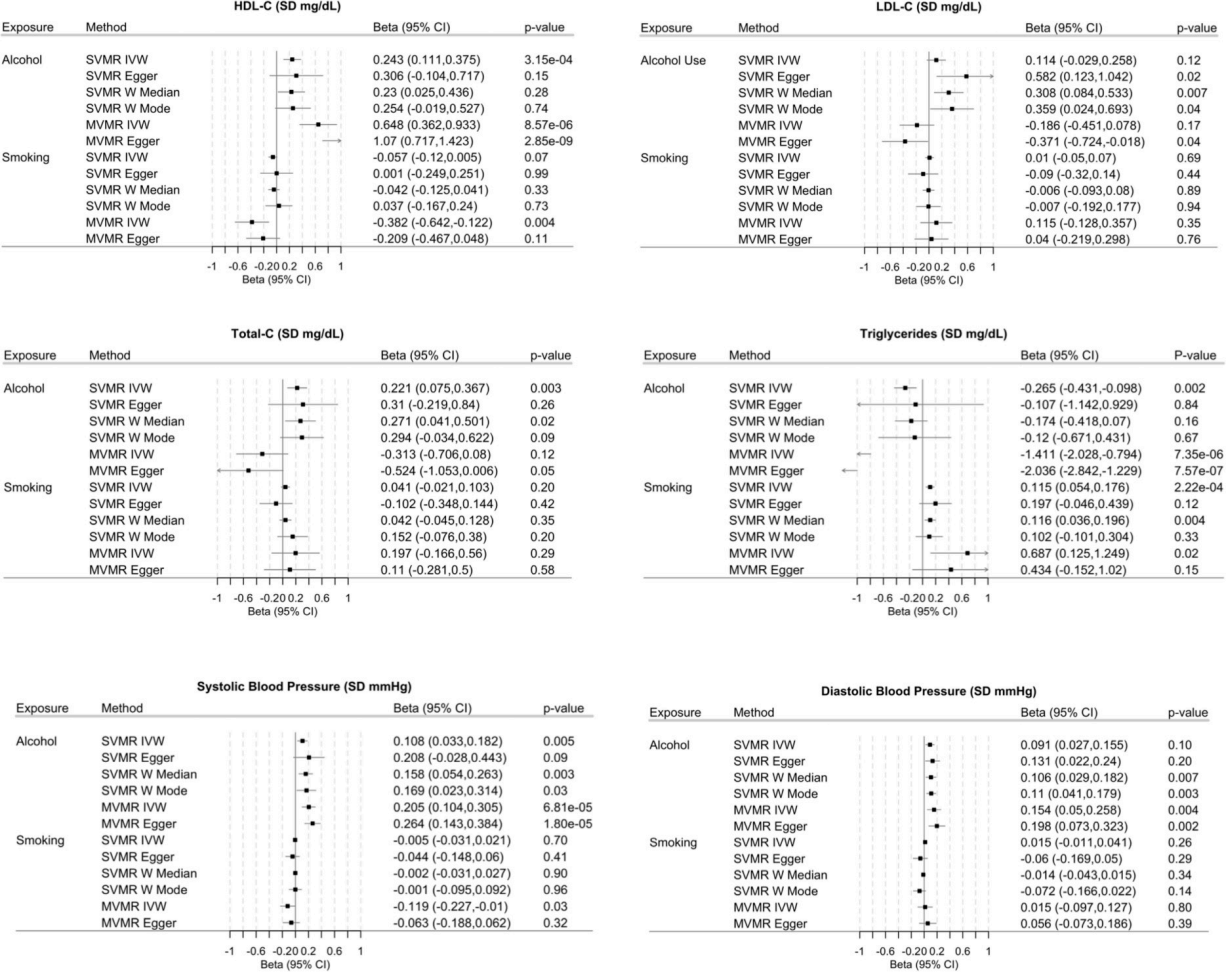
Associations of genetically predicted alcohol consumption and smoking with CVD risk factors. For alcohol, estimates are expressed per 1 SD increase in log-transformed weekly alcohol drinks consumed (MVMR adjusts for smoking). For smoking, estimates are expressed per 1 SD increase in the log odds of smoking regularly (MVMR adjusts for alcohol consumption). CI, confidence interval; CVD, cardiovascular disease; HDL-C, high-density lipoprotein cholesterol; IVW, inverse variance weighted; LDL-C, low-density lipoprotein cholesterol; MVMR, multivariable Mendelian randomization; SD, standard deviation; SNP, single nucleotide polymorphism.

**Fig 3:**
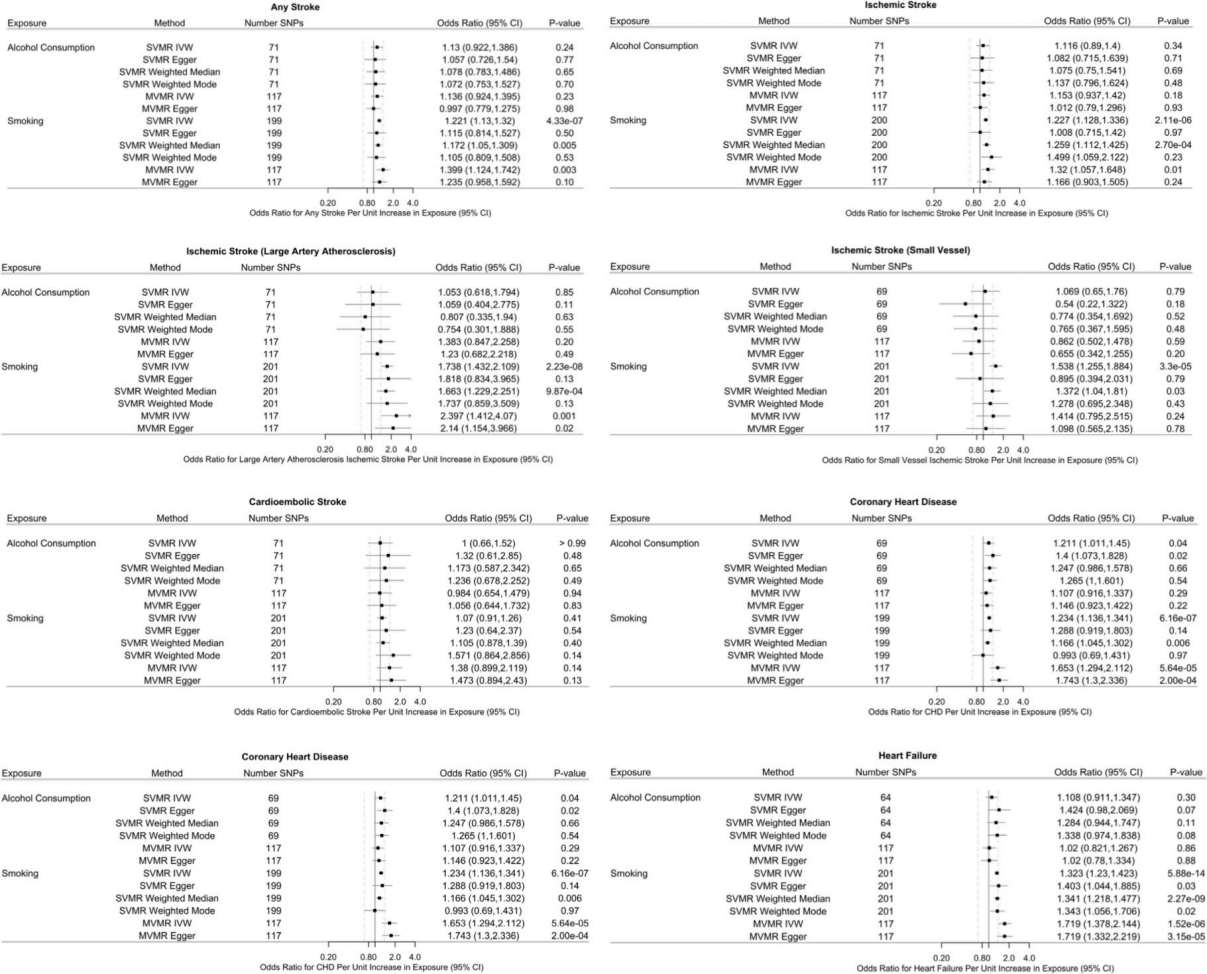
Associations of genetically predicted alcohol consumption and smoking with CVD outcomes. SVMR and IVW MVMR and MR–Egger estimates are shown as ORs. For alcohol, ORs are per 1 SD increase in log-transformed weekly alcohol drinks consumed (MVMR adjusts for smoking). For smoking, ORs are per 1 SD increase in the log odds of smoking regularly (MVMR adjusts for alcohol consumption). CI, confidence interval; CVD, cardiovascular disease; IVW, inverse variance weighted; MVMR, multivariable Mendelian randomization; OR, odds ratio; SD, standard deviation; SNP, single nucleotide polymorphism; SVMR, single-variable Mendelian randomization.

We generally look for those estimates (1) substantially agreeing in direction and magnitude across MR methods; (2) exceeding nominal significance (*P* = 0.05); (3) not indicating bias from horizontal pleiotropy (MR-PRESSO global *P* > 0.05, also MR–Egger intercept *P* > 0.05); and (4) for SVMR, indicating true causal direction (Steiger directionality test *P* < 0.05). MR results with test statistics both before and after outlier correction are presented in S3–S7 Tables. MR-PRESSO outlier corrected results are displayed in Figs 2 and 3. Estimates are presented as the IVW MR (with MR–Egger as the complementary method presented to draw inferences about causality), estimate (ß) (with 95% confidence interval (CI)), or, for the binary outcomes, as the odds ratios (ORs) (with 95% CI) per SD increase in the exposures (e.g., per SD increase of log-transformed alcoholic drinks per week).

We caution against interpreting the study findings based solely on the basis of a *P* value threshold [49,50]. We consider the wide range of outcomes to be a study strength and instead of constructing a “suggestive significance threshold” or ignoring potentially important findings that would be penalized given the wide number of CVD outcomes and risk factors included in this study (e.g., association of smoking on the risk for death due to cardiovascular causes (S7 Table)). Therefore, we report the conventional *P* value threshold of 0.05 and emphasize evidence strength based upon the estimate size, the 95% CI, and the *P* value.

## Results

### Impact of alcohol and smoking on CVD risk factors

Alcohol SVMR results are reported in S3 Table. In SVMR, we found genetic variants associated with increased alcohol associated with increasing GLGC HDL-C (beta 0.24, 95% CI, 0.11, 0.38, *P* value = 3.14 × 10^−4^) and lower TRG (beta −0.27, 95% CI, −0.43, −0.1, *P* value = 1.83 × 10^−3^) (Fig 2). These estimates were replicated in the UKB HDL-C (HDL-C beta 0.40, 95% CI, 0.04, 0.47, *P* value = 1.72 × 10^−28^; TRG beta −0.23, 95% CI, −0.30, −0.15, *P* value = 4.69 × 10^−10^) (S3 Table). Expanding to ApoA, ApoB, and lipoprotein A, genetically predicted alcohol consumption was associated with increased ApoA levels (beta 0.44, 95% CI, 0.38, 0.51, *P* value = 4.21 × 10^−35^) but decreased levels of ApoB (beta −0.09, 95% CI, −0.016, −0.017, *P* value = 0.016) and lipoprotein A (beta −0.067, 95% CI, −0.12, −0.011, *P* value = 0.019) (S3 Table). MR–Egger intercept analysis suggested some evidence of horizontal pleiotropy for these risk factors. Alcohol consumption was also associated with increased automated systolic BP measurement (beta 0.11, 95% CI, 0.03, 0.18, *P* value = 4.72 × 10^−3^) and automated diastolic BP measurement (beta 0.091, 95% CI, 0.027, 0.155, *P* value 5.24 × 10^−3^) (S3 Table). However, the Q-statistic suggest some residual heterogeneity for the estimates for both systolic and diastolic BP measurements.

Genetically predicted smoking SVMR results are reported in S4 Table. Notable smoking SVMR findings include inverse associations with the UKB HDL-C (beta −0.08, 95% CI, −0.11, −0.05, *P* value = 1.12 × 10^−8^), increases in both GLGC and UKB TRG (GLGC beta 0.12, 95% CI, 0.054, 0.18, 0.03, *P* value = 2.22 × 10^−4^; UKB beta 0.10, 95% CI, 0.01, 0.03, *P* value = 6.59 × 10^−12^), and both body surface area (BSA)-indexed left ventricular end-diastolic volume (beta −0.08, 95% CI, −0.14, −0.02, *P* value = 9.46 × 10^−3^) and BSA-indexed stroke volume (beta −0.08, 95% CI, −0.14, −0.03, *P* value = 0.005).

In MVMR analyses (Fig 2, S5 Table), genetically predicted alcohol retained its association with HDL-C, ApoA, and both BP readings. Notably, after accounting for smoking, alcohol was also associated with MRI-derived measurements of diastolic and systolic volumes, in addition to an increase in stroke volume. The SVMR and MVMR results were broadly replicated using the secondary genetic alcohol and smoking instruments (S4–S6 Tables).

### Impact of alcohol and smoking on CVD outcomes

Genetically predicted alcohol consumption was associated with increased risk for MI and CHD (MI OR 1.24, 95% CI, 1.03, 1.50, *P* value = 0.023; CARDioGRAMplusC4D CHD OR 1.21, 95% CI, 1.01, 1.45, *P* value = 0.038) (Fig 3, S3 Table). Genetically predicted alcohol consumption was also associated with the Neale Lab UKB CVD outcomes (stroke, stroke including and excluding SAH, and PAD); however, their estimate size was very small (e.g., OR PAD 1.005, 95% CI 1.002, 1.008, *P* value = 3.21 × 10^−4^) (S3 Table).

We found strong genetic evidence that genetically predicted smoking was associated with increased risk for HF (Heart Failure Molecular Epidemiology for Therapeutic Targets (HERMES) HF OR 1.32, 95% CI, 1.23, 1.42, *P* value = 5.88 × 10^−14^) and 4 of the ISGC stroke outcomes (e.g., ISGC ischemic stroke OR 1.74, 95% CI, 1.43, 2.11, *P* value = 2.23 × 10^−8^) (Fig 3, S4 Table). Smoking was also associated with increased for AF (OR 1.16, 95% CI, 1.10, 1.24, *P* value = 4.95 × 10^−7^), MI (OR 1.23, 95% CI, 1.12, 1.35, *P* value = 8.19 × 10^−6^), and all coronary-related CVD outcomes (e.g., CARDioGRAMplusC4D CHD OR 1.23, 95% CI, 1.14, 1.341, *P* value = 6.16 × 10^−7^; van der Harst CAD OR 1.30, 95% CI, 1.22, 1.39, *P* value = 6.95 × 10^−15^) (S4 Table); however, the Neale Lab UKB effect sizes were much smaller (e.g., Neale Lab UKB OR 1.02, 97% CI, 1.01, 1.02, *P* value = 2.48 × 10^−15^). SVMR smoking analyses showed some evidence of heterogeneity and residual pleiotropy (e.g., AF, van der Harst CAD, and CARDioGRAMplusC4D MI and CHD) (S4 Table).

When alcohol consumption and smoking initiation were examined together in MVMR (Fig 3, S5 Table), alcohol consumption maintained a direct impact on van der Harst CAD (OR 1.28, 95% CI, 1.07, 1.54, *P* value = 0.01), while its associations with CARDioGRAMplusC4D MI and CHD were diminished. Conversely, smoking initiation continued to demonstrate its association with ISGC stroke outcomes, HF, MI, and coronary-related CVD outcomes. Notably, after accounting for alcohol consumption, the relationship between genetically predicted smoking and the ISGC strokes, CARDioGRAMplusC4D MI and CHD (MI OR 1.84, 95% CI 1.43, 2.37, *P* value = 2.00 × 10^−6^; CHD OR 1.64, 95% CI, 1.28, 2.09, *P* value = 8.07 × 10^−5^), and HERMES HF and van der Harst CAD increased (e.g., HERMES OR 1.32 to 1.61, 95% CI, 1.32, 1.95, *P* value = 1.90 × 10^−6^; CAD OR = 1.66, 95% CI, 1.37, 2.00, *P* value = 1.39 × 10^−7^). Also, there was broadly less evidence of heterogeneity for MVMR compared to SVMR (S5 Table).

Full FinnGen results are in S6 and S7 Tables. We found genetically predicted alcohol consumption to only be associated with increased hypertension risk (OR 1.77, 95% CI, 1.28, 2.44, *P* value = 4.92 × 10^−4^), while smoking was associated with HF (OR 1.22, 95% CI, 1.07, 1.38, *P* value = 0.002), PAD (OR 1.63, 95% CI, 1.33, 1.98, *P* value = 1.38 × 10^−6^), CHD (OR 1.19, 95% CI, 1.02, 1.39, *P* value = 0.03), and atherosclerosis (OR 1.74, 95% CI, 1.39, 2.20, *P* value = 2.05 × 10^−6^) (S6 Table). Expanding to other FinnGen CVD outcomes, we found smoking was associated with death due to CVD causes (OR 1.19, 95% CI, 1.02, 1.39, *P* value = 0.027), transient ischemic attack (TIA; OR 1.27, 95% CI, 1.06, 1.51, *P* value = 0.01), diseases of the arteries, arterioles, and capillaries (DOAAC; OR 1.33, 95% CI, 1.14, 1.55, *P* value = 3.5 × 10^−4^), and cerebrovascular disease (OR 1.20, 95% CI, 1.01, 1.44, *P* value = 0.04) (S6 Table).

In MVMR of FinnGen CVDs, we found an impact of alcohol on AF (OR 1.87, 95% CI, 1.14, 3.08, *P* value = 0.01) adjusting for smoking, while smoking was still associated with HF (OR 1.67, 95% CI, 1.14, 2.46, *P* value = 0.008), PAD (OR 2.35, 95% CI, 1.38, 4.01, *P* value = 0.002), MI (OR 1.77, 95% CI, 1.10, 2.84, *P* value = 0.02), and cardiac death (OR 1.66, 95% CI, 1.02, 2.70, *P* value = 0.04) (S7 Table). Broadly, heterogeneity statistics were greater than *P* value > 0.05 for these findings with the exception of HF (heterogeneity *P* value = 0.003).

## Discussion

Our 2-sample MR design allowed us to estimate the total and direct impact of genetically predicted alcohol consumption and smoking on CVD risk factors and a wide range of CVD outcomes. In our SVMR, we also found evidence that alcohol consumption was associated with increased HDL-C, systolic and diastolic BP measurement, and the risk for stroke, hypertension, MI, CHD (as well as having a major CHD event), and PAD; however, the impact of alcohol consumption was attenuated in MVMR models incorporating smoking, after which alcohol consumption only maintained an association with increased risk for stroke, coronary atherosclerosis, and high BP. We were able to replicate in the FinnGen sample several smoking–CVD relationships identified in the other datasets, including HF, PAD, CHD, and atherosclerosis, whereas we found only a relationship between alcohol consumption on hypertension among FinnGen participants. Smoking–CVD replication using the FinnGen cohort strengthens causal inference in these relationships: we found, for example, approximately the same MVMR smoking–HF estimate sizes in both the HERMES (OR = 1.72) and FinnGen (OR = 1.67) samples. Concurrent evidence that the genetic predisposition for smoking had an impact on MRI-derived stroke volume further supports evidence for an impact of smoking on HF. Broadly, SVMR estimates were consistent in magnitude and direction across IVW, weighted median, and MR–Egger analyses. The MR–Egger estimate was substantially less precise, as is typically expected in MR genetic association studies, and the MR–Egger intercept was consistent with absence of pleiotropy [51]. Our estimates from conventional IVW MR were broadly consistent (within the IVW MR 95% CI but typically less precise) with estimates from the weighted median, weighted mode, and MR–Egger sensitivity analyses, which also strengthens causal inference.

Our findings support previous observational research showing a relationship between alcohol, smoking, and CVDs [5,10] and extend the literature to include more CVD risk factors and outcomes, including novel cardiac MRI data. Importantly, in our main analysis, we failed to find a cardioprotective relationship between alcohol use and CVD outcomes. Notably, alcohol was not associated with reduced CHD risk, which observational studies have reported to be the main CVD for which light-to-moderate alcohol consumption may be cardioprotective [52], suggesting that previously reported reductions in CVD risk due to other lifestyle differences that are associated with more light-to-moderate drinking patterns, such as healthier lifestyles [53], might be contributing to these observed relationships. Further, finding robust associations between genetically predicted alcohol consumption and increased HDL-C with no corresponding reduction in CVD risk calls into question the mechanism through which observational literature attributed any cardioprotective impact of light-to-moderate alcohol consumption [11]. Similarly, our results, when combined with traditional RCTs and MR studies that failed to find CVD risk reduction of increased HDL-C [54–56], suggest that there may be no cardioprotective role of alcohol consumption.

Using several BP outcomes, we found genetic evidence for an impact of alcohol on increased BP, which supports existing observational literature [57]. Given that it has been estimated that high BP accounts for approximately 54% of strokes and 47% of CHD [58], alcohol consumption might indirectly contribute to poor CVD outcomes by mechanism of increased BP.

In addition, we found evidence that genetically predicted that alcohol consumption was associated with increased stroke risk, which is consistent with MR estimates found among participants in the China Kadoorie Biobank sample; however, different MR estimate sizes (e.g., OR = 1.009 versus 1.27 among Kadoorie Biobank participants) support the hypothesized differential relationship between alcohol consumption and CVD risk across ethnic backgrounds [11,59]. East Asia has the highest prevalence of stroke worldwide [60] and generally, lower alcohol consumption behaviors than European countries [3]. In addition, survey data show that men consume 95% of all alcohol in China [61]. Therefore, caution is needed before extrapolating our findings to other populations, and our study highlights the need for population-specific MR analysis in elucidating the underlying alcohol–CVD relationship. We found less genetic evidence for an alcohol–stroke relationship than an early MR study that found that the *ADH1B* rs1229984 A-allele (associated with reduced alcohol consumption) was also associated with lower CHD and stroke risk [15]. However, they reported that rs1229984 was also associated with smoking, and therefore, omitting smoking may have influenced their results. They also found that rs1229984 was associated with increased educational attainment (EA), which more recent MR studies have shown to be causally linked with CVD risk [62]. Expanding upon their single-stroke variable, which may mask differential associations between alcohol and stroke subtypes [8], is another potential reason for the disparity. Further, finding a concurrent relationship between alcohol and BP, which increases stroke risk [63], suggests a potential mechanism through which alcohol may impact stroke risk.

Our findings also support an inference of the causal role of alcohol consumption in increasing HDL-C as reported in previous MR studies [13,19] and extends the existing literature by finding the MR estimate robust to models accounting for smoking behavior. Replicating the MR estimate using an additional alcohol consumption dataset as well as with the recently published and largest-to-date HDL-C data derived from the UKB participants, with a sample size almost 3 times greater than the GLGC lipid data, also extends the literature. While we were unable to evaluate the possible impact of heavy alcohol consumption on HDL-C levels, we recently reported a cross-sectional study finding very heavy alcohol consumption (more than 10 standard drinks for men and more than 8 standard drinks for women) to be associated with increased HDL-C [64], which suggests that alcohol may impact HDL-C levels across a range of alcohol consumption levels.

Consistent with the observational literature, we found genetic evidence of a causal relationship between smoking, independent of alcohol use, on increased risk for MI, CHD (including the risk for a major CHD event), PAD, stroke, and ischemic stroke (large artery atherosclerosis) [5]. Our estimates showing that smoking was associated with 48% and 30% increased risk for MI and CHD, respectively, are similar to estimates from observational and smoking cessation studies [65], and we confirm findings from an MR study that found a smoking instrument constructed from multiple smoking behaviors increased CHD and MI risk [62] by replicating their results using a separate smoking instrument accounting for alcohol consumption. In addition, using the death due to cardiovascular causes outcome in the FinnGen dataset (5,569 cases/130,069 controls), we found some evidence for a causal relation of smoking initiation on CVD-related death (SVMR OR = 1.18, MVMR OR = 1.68), which aligns with a meta-analysis finding smoking cessation associated with a 36% reduced mortality risk (notably, these MR estimate sizes are similar in magnitude to other prevention strategies, including statins and ß-blockers [66]). While an increased awareness and understanding of the adverse health consequences associated with smoking has decreased the prevalence of current smoking, smoking continues to be a major cardiovascular hazard [67]. For example, in the United States, 44 million adults still smoke, suggesting that if the association between smoking and CVD risk is causal, our results may be useful to further inform continued clinical practice and smoking prevention program development, which would substantially reduce the morbidity and mortality related to CVD.

Supporting the observational literature [5], we found genetic evidence for a causal relationship between smoking and decreased levels of HDL-C and increased levels of TRG. Conversely, our findings do not support Åsvold and colleagues’ MR analysis of the Norwegian HUNT Study cohort showing higher HDL-C and no impact on TRG using a single-SNP instrument (rs1051730 located in *CHRNA3*) [68]. However, associations between rs1051730 and HDL-C were not significantly different by smoking status in the HUNT cohort, and even the authors cautioned interpretation of their HDL-C finding [68]. Smoking has been shown to alter lipid metabolism enzymes, which would alter HDL-C levels [69]. Further, HDL-C particles can be rendered nonfunctional due to oxidative modifications from smoking, which suggests that smoking may impact HDL-C quantity and function [69].

Our study has several strengths. We use a 2-sample MVMR design, the most appropriate design given the strong correlation between alcohol and tobacco use, yielding adjusted estimates of each exposure on CVD risk factors and disease events [70]. Another major strength is the use of the FinnGen data, used to both increase the number of CVDs evaluated and replicate several of the smoking-related findings: To our knowledge, this study evaluates a wider range of CVD outcomes and risk factors of any MR study to date. Similarly, broadly replicating results using secondary alcohol consumption and smoking initiation datasets also improves causal inference. We also used multiple MR methods, each relying on orthogonal assumptions that assess the validity of the MR assumptions, provides confidence in result robustness, and strengthens causal inference [29]. Finally, while previous MR studies only used a combined stroke variable, we used multiple stroke subtypes, which, given some observational studies reporting a differential association of alcohol by stroke subtype [8], may help elucidate the alcohol–stroke relationship.

Limitations are also noted. While our sensitivity analyses incorporating complementary MR methods failed to find evidence of pleiotropy, it is still possible that confounding and pleiotropy may be present (for example, the included SNPs in the alcohol consumption instrument may impact CVD risk through other pathways), which would potentially bias the results [45]. Weak instrument bias may also exist; however, we chose the SNPs for the instruments using stringent criteria (conventional genome-wide significance (*P* value < 5 × 10^−8^), excluding SNPs within a 10,000 kb window, LD R^2^ > 0.001, etc.). Also, SNP F statistics, which assess the instrument strength, all exceeded the conventional cutoff (>10). In addition, the binary nature of our smoking variable and the majority of CVD outcomes make it difficult to interpret specific causal relationships [71]. Therefore, it is not possible to directly compare the resultant MR estimates with corresponding estimates from observational data; however, these MR estimates do provide a valid test of the possible causal null hypothesis [72]. We emphasize again that the evidence strength should be evaluated based upon the magnitude and the CI of the estimate. We have included results that would not have survived correction for multiple comparisons so as not to overlook possible clinically relevant findings. However, these findings would require future studies to strengthen evidence of an association.

We caution against interpreting the study findings based solely on the basis of a *P* value threshold [49,50]. We consider the wide range of outcomes to be a study strength and instead of constructing a “suggestive significance threshold” or ignoring potentially important findings that would be penalized given the wide number of CVD outcomes and risk factors included in this study (e.g., effect of smoking on the risk for death due to cardiovascular causes) (S7 Table). Therefore, we report the conventional *P* value threshold of 0.05 and emphasize evidence strength based upon the effect size, the 95% CI, and the *P* value.

As with previous alcohol literature, alcohol consumption patterns are variable, and the exposure variables may be either under- or overreported [20]. Carter and colleagues found that smoking mediates the relationship between education and CVD risk [62], and we recently reported that a 2-sample MR finding EA affects how alcohol is consumed—with increased EA increasing the frequency of consuming alcohol with meals and consuming alcohol more frequently overall, but decreasing the risk of binge drinking (≥6 drinks per occasion) [73]. Therefore, future studies using cohorts with heavier reported consumption or incorporating dietary patterns associated with different drinking typologies would further elucidate the alcohol–CVD relationship. Future work—when an alcohol consumption variable that accounts for drinking patterns becomes available—is necessary. We examined total alcohol consumption among participants in the UKB, who have been reported to be more educated, with healthier lifestyles, and fewer health problems than the UK population [74], which may limit the applicability to other populations. We were also unable to evaluate other hazardous consumption patterns, which may have additional adverse CVD consequences [75]. Further, while we fail to find evidence of a cardioprotective impact for alcohol, it is possible that our alcohol consumption instrument may not be sensitive enough to account for distinct and potentially different in their relationship with CVDs. In addition, current summary-level MR methods are poorly suited to assess the potentially nonlinear relationship between alcohol consumption and CVD risk, so future individual-level MR studies are warranted.

The included GWASs are from survey participants of predominantly European ancestry, and given observational evidence suggesting different associations between alcohol and CVDs exists across racial and ethnic groups [11], caution may be advised in extrapolating these findings to other racial and ethnic groups. Further, some GWS exposure SNPs, or their proxies, were not available in every outcome GWAS used in this study, suggesting reduced variance captured with the remaining SNPs [29]. Another limitation is the overlap of the UKB participants in many of the exposure and outcome datasets, which could bias the estimates [76]; however, any bias would likely be minimal [76], and it has been also recently shown that 2-sample MR methods may be safely used in single samples provided the data are derived from large biobanks (i.e., the UKB) [77]. Finally, there also existed some overlap in the cohorts used in the outcome CVD sample (e.g., the van de Harst CAD GWAS contained the UKB and CARDIoGRAMplusC4D CHD participant data), which would not bias the findings; however, these samples are not independent, and their corresponding findings do not represent replication.

In conclusion, we found genetic evidence for adverse impact of alcohol on several CVD outcomes. Further, alcohol retained its association with models accounting for genetically predicted smoking behavior on increased risk for CHD, HDL-C, and BP. Conversely, we failed to find evidence supporting a cardioprotective role of alcohol consumption on CVD risk factors and outcomes, suggesting previous observational studies identifying a cardioprotective relation of light-to-moderate alcohol consumption may be due to confounding factors such as healthier lifestyles. We also found evidence that smoking may have an impact on the risk for HF, MI, CHD, and several other CVD outcomes. Notably, in MVMR, genetically predicted smoking retained an impact on increased risk for MI, CHD, PAD, and stroke. While future studies further examining the role alcohol consumption patterns are necessary when the data become available, these findings may also help inform the public health debate regarding the alcohol–smoking–CVD relationship.

## Supporting information

S1 STROBE Checklist (DOCX)

S1 Example Code (DOCX)

S1 Table Phenotype source and description. (XLSX)

S2 Table Genetic instruments for alcohol consumption harmonized with and smoking initiation/ever-smoker status. (XLSX)

S3 Table SVMR results of primary and secondary alcohol consumption exposures. (XLSX)

S4 Table SVMR results of primary and secondary smoking exposures. (XLSX)

S5 Table MVMR results of primary and secondary alcohol consumption and smoking exposures. (XLSX)

S6 Table SVMR results of primary alcohol consumption and smoking exposures on FinnGen Datafreeze 3 CVD outcomes. (XLSX)

S7 Table MVMR results of primary alcohol consumption and smoking exposures on FinnGen R3 disease endpoints. (XLSX)

## Abbreviations

AF: atrial fibrillation
AFGen: Atrial Fibrillation Consortium
ApoA: apolipoprotein A
ApoB: apolipoprotein B
BP: blood pressure
BSA: body surface area
CAD: coronary artery disease
CARDIoGRAMplusC4D: Coronary ARtery DIsease Genome-wide Replication and Meta-analysis (CARDIoGRAM) plus The Coronary Artery Disease (C4D) Genetics
CHD: coronary heart disease
CI: confidence interval
CVD: cardiovascular disease
DOAAC: diseases of the arteries, arterioles, and capillaries
DPW: drinks per week
EA: educational attainment
EHRs: electronic health records
GLGC: Global Lipids Genetics Consortium
GWAS: genome-wide association study
HDL-C: high-density lipoprotein cholesterol
HERMES: Heart Failure Molecular Epidemiology for Therapeutic Targets
HF: heart failure
HUNT: Trøndelag Health Study
ISGC: International Stroke Genetics Consortium
IVW: inverse variance weighted
LD: linkage disequilibrum
LDL-C: low-density lipoprotein cholesterol
MI: myocardial infarction
MGI: Michigan Genomics Initiative
MR: Mendelian randomization
MRC-IEU: Medical Research Council Integrative Epidemiology Unit
MRI: magnetic resonance imaging
MR-PRESSO: Mendelian randomization pleiotropy residual sum and outlier
MV: multivariable
MVMR: multivariable Mendelian randomization
OR: odds ratio
PAD: peripheral artery disease
PHESANT: PHEnome Scan Analysis Tool
RCT: randomized control trial
SAH: subarachnoid hemorrhage
SD: standard deviation
STROBE: Strengthening the Reporting of Observational Studies in Epidemiology
SV: single-variable
SVMR: single-variable Mendelian randomization
TAG, Tobacco: Alcohol and Genetics
TC: total cholesterol;TIA, transient ischemic attack
TRG: triglycerides
UKB: UK Biobank
