# Supplementary material for: Evaluating the relationship between alcohol consumption, tobacco use, and cardiovascular disease: A multivariable Mendelian randomization study
Source: PLoS Med. 2020 Dec 4;17(12):e1003410. doi: 10.1371/journal.pmed.1003410 (PMC7717538; doi:10.1371/journal.pmed.1003410)
Supplement: S1 Example Code — (DOCX) [file pmed.1003410.s002.docx]

#example single variable Mendelian randomization code for alcohol consumption on a Finngen

#open TwoSampleMR

library(TwoSampleMR)

#import alcohol consumption FinnGen dataset, which can be accessed at https://www.finngen.fi/en

#Note: The alcohol consumption instrument datafile is in the S3 Table

#Format to create TwoSampleMR outcome dataset

FG_kcardiac_gwas <- finngen_r3_I9_K_CARDIAC

FG_kcardiac_gwas$SNP <- FG_kcardiac_gwas$V5

FG_kcardiac_gwas$effect_allele <- FG_kcardiac_gwas$V4

FG_kcardiac_gwas$other_allele <- FG_kcardiac_gwas$V3

FG_kcardiac_gwas$beta <- FG_kcardiac_gwas$V8

FG_kcardiac_gwas$se <- FG_kcardiac_gwas$V9

FG_kcardiac_gwas$pval <- FG_kcardiac_gwas$V7

#extract alcohol consumption genetic variants from FinnGen dataset

liuDPW_FG_out <- subset(FG_kcardiac_gwas, FG_kcardiac_gwas$SNP == "rs705687" | FG_kcardiac_gwas$SNP =="rs58107686" | FG_kcardiac_gwas$SNP == "rs12088813" | FG_kcardiac_gwas$SNP =="rs5024204" | FG_kcardiac_gwas$SNP =="rs10753661" | FG_kcardiac_gwas$SNP == "rs28680958" | FG_kcardiac_gwas$SNP =="rs823114" | FG_kcardiac_gwas$SNP =="rs77165542" | FG_kcardiac_gwas$SNP =="rs1260326" | FG_kcardiac_gwas$SNP =="rs13383034"

| FG_kcardiac_gwas$SNP =="rs13032049" | FG_kcardiac_gwas$SNP =="rs828867" | FG_kcardiac_gwas$SNP == "rs11692435" | FG_kcardiac_gwas$SNP =="rs13024996"| FG_kcardiac_gwas$SNP =="rs72859280" | FG_kcardiac_gwas$SNP =="rs56337305" | FG_kcardiac_gwas$SNP =="rs13094887" | FG_kcardiac_gwas$SNP =="rs62250685" | FG_kcardiac_gwas$SNP =="rs9838144" | FG_kcardiac_gwas$SNP =="rs2011092"

| FG_kcardiac_gwas$SNP == "rs6787172" | FG_kcardiac_gwas$SNP =="rs3748034" | FG_kcardiac_gwas$SNP =="rs11940694" | FG_kcardiac_gwas$SNP =="rs4501255" | FG_kcardiac_gwas$SNP =="rs1229984" | FG_kcardiac_gwas$SNP =="rs2165670" | FG_kcardiac_gwas$SNP =="rs13107325" | FG_kcardiac_gwas$SNP =="rs4690727" | FG_kcardiac_gwas$SNP =="rs12651313" | FG_kcardiac_gwas$SNP =="rs4916723"

| FG_kcardiac_gwas$SNP =="rs12655091" | FG_kcardiac_gwas$SNP =="rs55872084" | FG_kcardiac_gwas$SNP =="rs6460047" | FG_kcardiac_gwas$SNP =="rs10236149" | FG_kcardiac_gwas$SNP =="rs35034355" | FG_kcardiac_gwas$SNP =="rs6951574" | FG_kcardiac_gwas$SNP =="rs13250583" | FG_kcardiac_gwas$SNP =="rs1217091" | FG_kcardiac_gwas$SNP =="rs28601761" | FG_kcardiac_gwas$SNP =="rs55932213"

| FG_kcardiac_gwas$SNP == "rs10978550" | FG_kcardiac_gwas$SNP == "rs7074871" | FG_kcardiac_gwas$SNP =="rs17665139" | FG_kcardiac_gwas$SNP =="rs7950166" | FG_kcardiac_gwas$SNP =="rs11030084" | FG_kcardiac_gwas$SNP =="rs56030824" | FG_kcardiac_gwas$SNP =="rs10750025" | FG_kcardiac_gwas$SNP =="rs4938230" | FG_kcardiac_gwas$SNP =="rs682011" | FG_kcardiac_gwas$SNP =="rs12795042"

| FG_kcardiac_gwas$SNP == "rs3809162" | FG_kcardiac_gwas$SNP =="rs10506274"| FG_kcardiac_gwas$SNP =="rs4842786" | FG_kcardiac_gwas$SNP =="rs500321" | FG_kcardiac_gwas$SNP =="rs1123285" | FG_kcardiac_gwas$SNP =="rs28929474" | FG_kcardiac_gwas$SNP =="rs2472297" | FG_kcardiac_gwas$SNP =="rs12907323" | FG_kcardiac_gwas$SNP =="rs17177078" | FG_kcardiac_gwas$SNP =="rs378421"

| FG_kcardiac_gwas$SNP =="rs62044525" | FG_kcardiac_gwas$SNP =="rs1104608" | FG_kcardiac_gwas$SNP =="rs4548913" | FG_kcardiac_gwas$SNP == "rs3803800" | FG_kcardiac_gwas$SNP =="rs2854334" | FG_kcardiac_gwas$SNP =="rs10438820" | FG_kcardiac_gwas$SNP =="rs9950000" | FG_kcardiac_gwas$SNP =="rs4092465" | FG_kcardiac_gwas$SNP =="rs281379" | FG_kcardiac_gwas$SNP =="rs4815364"

| FG_kcardiac_gwas$SNP =="rs9607814")

# format outcome datafile

liuDPW_FG_out <- TwoSampleMR::format_data(liuDPW_FG_out, type="outcome")

liuDPW_FG_out$outcome = "FG1_kcardiac"

# harmonise exposure and outcome data

dat_liuDPW_kcardiac <- TwoSampleMR::harmonise_data(exposure_dat = LiuDPW_exp_dat_0618, outcome_dat =liuDPW_FG_out, action = 1 )

#Perform single variable Mendelian randomziation

res <- TwoSampleMR::mr(dat_liuDPW_kcardiac)

res

mrhet <- TwoSampleMR::mr_heterogeneity(dat_liuDPW_kcardiac)

mrhet

mrp <- TwoSampleMR::mr_pleiotropy_test(dat_liuDPW_kcardiac)

mrp

stdir <- TwoSampleMR::directionality_test(dat_liuDPW_kcardiac)

stdir

#import MRPRESSO package

library(MRPRESSO)

#identify outler SNPs (if necessary)

mr_presso(BetaOutcome = "beta.outcome", BetaExposure = "beta.exposure", SdOutcome = "se.outcome",

SdExposure = "se.exposure", OUTLIERtest = TRUE, DISTORTIONtest = TRUE,

data = dat_liuDPW_kcardiac, NbDistribution = 2000, SignifThreshold = 0.3)

#created harmonized dataset with outlier SNPs removed, if necessary

dat_liuDPW_kcardiac_oc <- dat_liuDPW_kcardiac[c(-62),]

#perform single variable Mendelian randomization on new, outlier corrected dataset, as necessary.

res <- TwoSampleMR::mr(dat_liuDPW_kcardiac_oc)

res

mrhet <- TwoSampleMR::mr_heterogeneity(dat_liuDPW_kcardiac_oc)

mrhet

mrp <- TwoSampleMR::mr_pleiotropy_test(dat_liuDPW_kcardiac_oc)

mrp

stdir <- TwoSampleMR::directionality_test(dat_liuDPW_kcardiac_oc)

stdir
